# Design and Sensing Applications of Eutectogels: A Review

**DOI:** 10.3390/ma19061059

**Published:** 2026-03-10

**Authors:** Ke Zhang, Yan Huang, Jiangxue Han, Zhangpeng Li, Jinqing Wang, Shengrong Yang

**Affiliations:** 1 State Key Laboratory of Solid Lubrication, Lanzhou Institute of Chemical Physics, Chinese Academy of Sciences, Lanzhou 730000, China; zhangke231@licp.cas.cn (K.Z.); huangyan@licp.cas.cn (Y.H.); hanjiangxue@licp.cas.cn (J.H.); sryang@licp.cas.cn (S.Y.); 2 Center of Materials Science and Optoelectronics Engineering, University of Chinese Academy of Sciences, Beijing 100049, China; 3 Qingdao Center of Resource Chemistry & New Materials, Qingdao 266000, China

**Keywords:** deep eutectic solvents, eutectogels, material selection, sensing mechanisms, applications

## Abstract

Deep eutectic solvent (DES), when used as the continuous phase of eutectogels, can significantly improve their electrical and mechanical properties due to its excellent conductivity, freeze resistance and chemical stability. The development of eutectogels effectively solves the key limitations of traditional hydrogels and organogels, such as low-temperature freezing, high-temperature volatilization, and organic solvent leakage. It also realizes the collaborative optimization of environmental friendliness and comprehensive performance, which makes it show broad application prospects in the field of flexible sensing. This review summarizes the design principles, material selection, sensing mechanisms, and flexible sensing applications of eutectogels. By examining the design of eutectogels, the selection of DES, and the synthesis of the gel network, it provides a theoretical basis for the development of eutectogel-based sensor devices. A detailed description of the sensing mechanism is provided to elucidate the signal generation and transition in eutectogels toward the purpose of the practical applications. Finally, the application prospects of eutectogels for high-performance sensors and detection devices are discussed. Additionally, we provide a theoretical support for their structural design, performance optimization, and practical application.

## 1. Introduction

Gel-based flexible sensing materials, characterized by excellent flexibility, biocompatibility, and functional integration, have been widely applied in health monitoring [1], human–computer interaction [2], wearable electronics [3], and artificial prosthetics [4]. However, traditional gel materials using water or organic solvents as the continuous phase are susceptible to structural degradation due to evaporation [5], freezing, and leakage of solvents, which diminishes the accuracy of sensing signals and compromises their ability to fulfill the requirements for continuous and stable sensing. Ionogels [6], featuring ionic liquids as the continuous phase, have effectively addressed the issues of low-temperature freezing and solvent leakage associated with aforementioned conventional gels. However, the complex and costly synthesis of ionic liquids greatly limits the broader application of ionogels. Recently, eutectogels, using deep eutectic solvents (DES) as the dispersion medium, have emerged and have attracted increasing attention, as they possess key advantages, including high ionic conductivity [7], exceptional environmental stability [8], and prominent mechanical properties. They not only overcome the inherent shortcomings of traditional gels but also broaden the emerging application scope of gel systems.

DES are homogeneous liquid mixtures featuring a drastically depressed freezing point, and which are formed via hydrogen bonding interactions between hydrogen bond acceptors (HBAs) and hydrogen bond donors (HBDs) in specific molar ratios. Such strong hydrogen bonding interaction between HBA and HBD disrupts the original crystal structures of the individual components, forming a mixture with lower lattice energy, as first reported by Abbott et al. [9] in 2003. They combined choline chloride (a quaternary ammonium salt) with urea in a molar ratio of 1:2 to yield an eutectic mixture. Owing to the hydrogen bonding interactions between urea molecules and chloride ions, the freezing temperature of this mixture is only 12 °C, which is significantly lower than that of the raw materials choline chloride (302 °C) and urea (133 °C) [9]. HBAs are primarily quaternary ammonium salts, while HBDs boast a diverse array of options, encompassing amides [10,11], carboxylic acids [12,13], polyols [14,15], carbohydrates [16] and phenols. The abundant sources of DES provide a way to precisely control their physicochemical properties.

Eutectogels fabricated with DES as the continuous phase are semi-solid materials with a solid-like morphology and plasticity, and which are stabilized and entrapped by three-dimensional cross-linked polymer networks or small-molecule self-assembled [13]. As a functional composite system integrating DES and gel materials, by adjusting the hydrogen bond strength and density in DES, it is organically combined with the hydrogen bond active groups of the eutectogel network, dynamically adjusting the mechanical properties of the eutectogels. Meanwhile, the ionic conductivity [17] characteristics of DES endow the eutectogels with excellent ionic conductivity. The free cations and anions dissociated from DES, are dispersed in the three-dimensional network of the eutectogels. Under the stimulation of an electric field or external force, their directional migration generates an ionic current, constituting the conductive basis of the gel. The excellent interfacial properties of DES serve as the key origin of the interfacial adhesion force of the eutectogels. By choosing different types of functional groups in DES, their interfacial adhesion behavior is directionally regulated, significantly enhancing the adhesion performance of eutectogels. Eutectogels leverage the synergistic combination of properties from both materials. They not only retain the intrinsic core properties of DES, including facile preparation, exceptional stability, tunable physicochemical properties, environmental benignity, and outstanding ionic conductivity [18,19,20], but also exhibit the unique viscoelasticity inherent to gel. This advantageous combination of dual properties endows them with the unique competitiveness of rapid response and high sensitivity in sensing applications, establishing them as key materials for advancing the field of gel-based sensors beyond traditional limitations. Eutectogels, renowned for their tunable mechanical properties, outstanding environmental stability, and multi-stimulus responsiveness, are progressively reshaping the frontiers of flexible sensing technologies.

At present, many reviews have systematically discussed the component composition and synthesis preparation methods of DES and eutectogels [18,21,22,23]. However, there is still a lack of systematic summaries and overviews regarding the design of eutectogels as flexible sensors and the regulatory role of DES components on their sensing performance. This review systematically summarizes the research progress in eutectogel-based flexible sensors. First of all, it details the critical influence of rational DES selection and the structural design of gel networks on their flexible sensing performance; notably, the selection of compatible DES components and the precise modulation of gel network strength are foundational to realize high-performance flexible sensors. Subsequently, we summarize three distinct sensing mechanisms that meet the requirements of diverse application scenarios through differentiated signal conversion principle, including resistive sensing, capacitive sensing, and triboelectric sensing. Moreover, the applications of eutectogel-based sensors in different scenarios are analyzed and discussed. Finally, future prospects of eutectogel-based sensors are proposed.

## 2. The Design of Eutectogels

**Figure 1:**
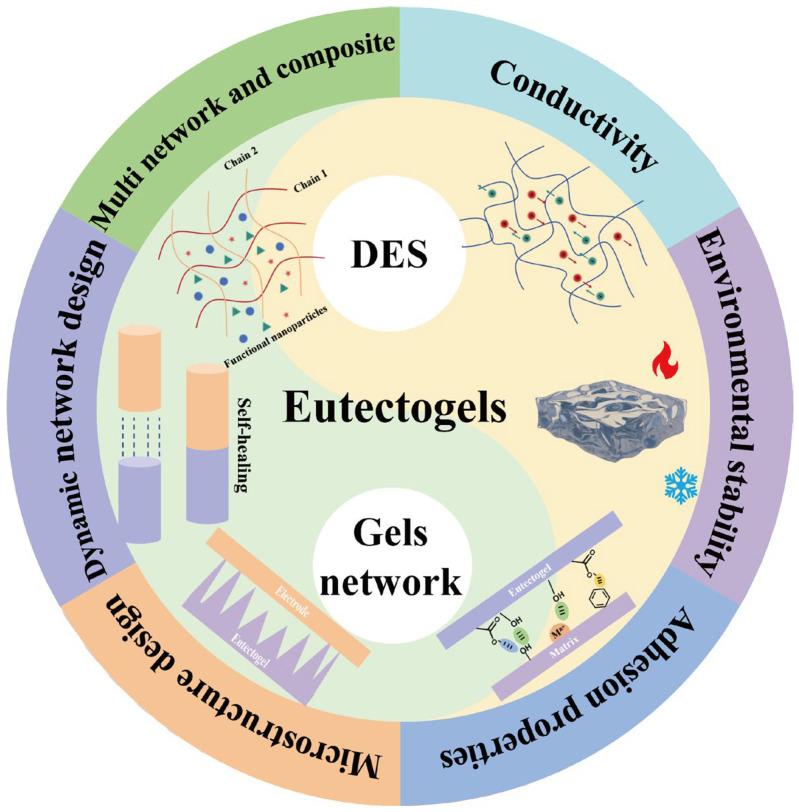
An overview of the structural composition and properties of eutectogels.

In the design and preparation of eutectogels, DES is used as a continuous phase instead of traditional aqueous organic solvent, which has significant advantages for performance regulation. The conductivity and environmental stability of eutectogels can be adjusted by selecting the type and regulating the molar ratio of HBA and HBD [19,20,24]. As shown in Table 1, by selecting different types of DES and gel networks, key parameters, such as conductivity, environmental stability and the adhesion of the eutectogel, have a significant impact on properties of eutectogels. Therefore, the selection and design of DES is of great significance for the sensing performance of the eutectogel. At the same time, as the core framework of sensor devices, the gel network can be optimized in terms of its structure and performance through a variety of strategies. Microstructural design can improve the contact area and response rate [25], dynamic network construction can endow with self-healing capability to the eutectogels, and the multi-network composites can improve their mechanical strength [26]. In Table 2, the design factors of eutectogel and its positive effect on sensing performance are explained in detail and the advantages and disadvantages are presented. These optimization strategies jointly realize the collaborative improvement of the sensing sensitivity, self-healing performance, and mechanical properties. Therefore, the selection of DES and the refined design of gel network are fundamental prerequisites for enabling eutectogels to satisfy stable sensing requirements across diverse scenarios. The functions of the key components of eutectogel are summarized in Figure 1.

### 2.1. Selection of DES in Eutectogels

**Figure 2:**
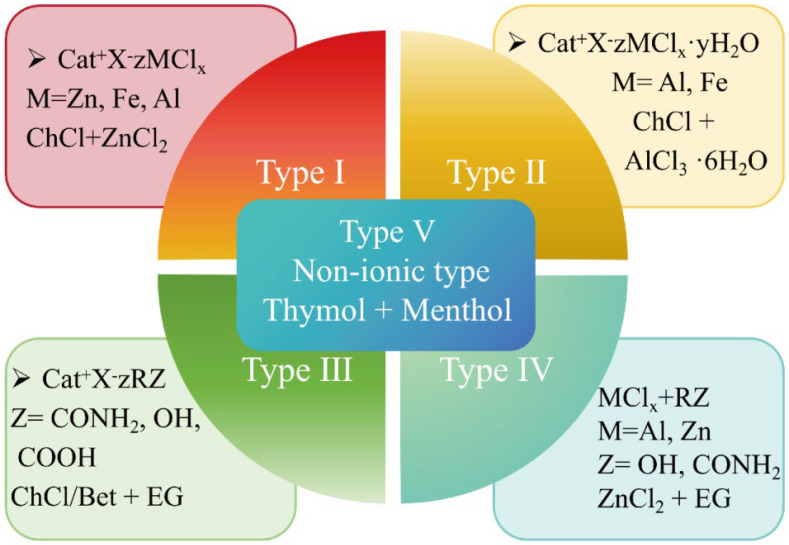
Five types of DES and their composition.

Since the first report by Abbott et al. [9], five types of DES have been developed, as shown in Figure 2 with their respective compositions [47,48,49]. The selection of a specific DES exerts a profound impact on the performance of eutectogels. This section elaborates on the influence of DES selection on the conductivity, stability, and interfacial properties of eutectogels, providing a theoretical foundation for designing their sensing performance.

#### 2.1.1. Conductivity

The ionic conductivity property of DES is the key source of the electrical performance of ionic conductive eutectogels. The freely dissociated positive and negative ions of DES, acting as conductive carriers, are dispersed in the three-dimensional network of the eutectogels. Under electric field or external force, they undergo directional migration to generate an ionic current, constituting the conductive basis of the gel. By selecting HBA with different dissociation degrees, adjusting the molar ratio of HBA, and by introducing low-viscosity HBD or a small amount of inorganic salts, the directional tuning of DES conductivity and electrochemical window can be achieved. High dissociation degree HBA, high HBA ratio, and low-viscosity HBD are all conducive to enhancing the conductivity of DES.

Among the five types of DES, the HBAs of type I and type II are organic salts [36], and their HBDs consist primarily of anhydrous metal halides and metal halides. The presence of metal ions significantly enhances the conductivity of the resulting eutectogels [19,27]. However, the limited selection of available HBAs hinders the expansion of these types. Furthermore, despite the enhanced conductivity, the instability of HBDs, such as the hygroscopic nature of anhydrous metal chlorides, has led to their limited use in eutectogel synthesis.

Type III DES is the most commonly used DES in eutectogel sensing, owing to the broad availability and diverse selection of HBAs and HBDs. The HBAs are mainly organic salts (e.g., choline chloride [50,51,52,53], betaine [54], etc.). The range of HBDs is even more diverse, mainly including alcohols, such as ethylene glycol [55], 1,2-propanediol (PG) [56], glycerol [57]; amides, such as urea [58] and acrylamide [59]; and carboxylic acids, such as acrylic acid [60], lactic acid [61], and phytic acid [62]. Different from type I and type II DES containing metal ions, type III DES does not require metal salts, and can be prepared by mixing the HBA and HBD in a specific molar ratio followed by heating.

Type III DES, as the mainstream category of ionic DES, balances conductivity effectiveness and practical stability. Although it lacks the extremely high conductivity that is contributed by metal ions, it realizes ionic conductivity through organic anion and cation ions dissociated from quaternary ammonium salts. Its conductivity stability and long-term consistency are superior. Meanwhile, its dense hydrogen bond network can naturally achieve a synergy between the conductive performance and the mechanical properties of the eutectogels, which avoids the deterioration of the mechanical properties of the eutectogels and accompanies high conductivity. This meets the conductive requirements of flexible sensors and other devices.

Type IV DES uses metal halides as HBAs, with the primary HBDs being amides and alcohols [35,37,63]. However, the strong interactions among metal chloride ions result in an abnormal elevation in solvent viscosity. This high viscosity restricts the solubility of raw materials and the ion mobility during sensing. Consequently, even at high concentrations of metal ions, the conductivity of eutectogels is significantly reduced [64]. These limitations are more pronounced than those of other types and prove difficult to mitigate through simple optimization. Type V DES is non-ionic and is composed of non-ionic HBDs and non-ionic HBAs [65,66]. The concept of a Type V DES is relatively new, first proposed by Coutinho et al. [67], and represents the situation whereby most non-ionized systems only achieve weak conductivity through molecular dipole moments, and are nearly insulating. A few weak ionic systems have a low degree of proton dissociation and extremely low carrier concentration, resulting in a conductive performance far lower than that of types I, II, III, and IV ionic DES. They fail to provide effective conductivity for eutectogels and find it difficult to meet the conductivity requirements of medium- and high-sensitivity flexible sensors. Moreover, the controllability of conductivity is extremely poor. Due to the lack of effective conductive carriers, adjusting parameters such as the types and ratios of HBA/HBD only allows for a modest variation in molecular dipole moments, and cannot achieve precise control of conductivity. There is little room for customized design of conductive properties.

#### 2.1.2. Environmental Stability

**Figure 3:**
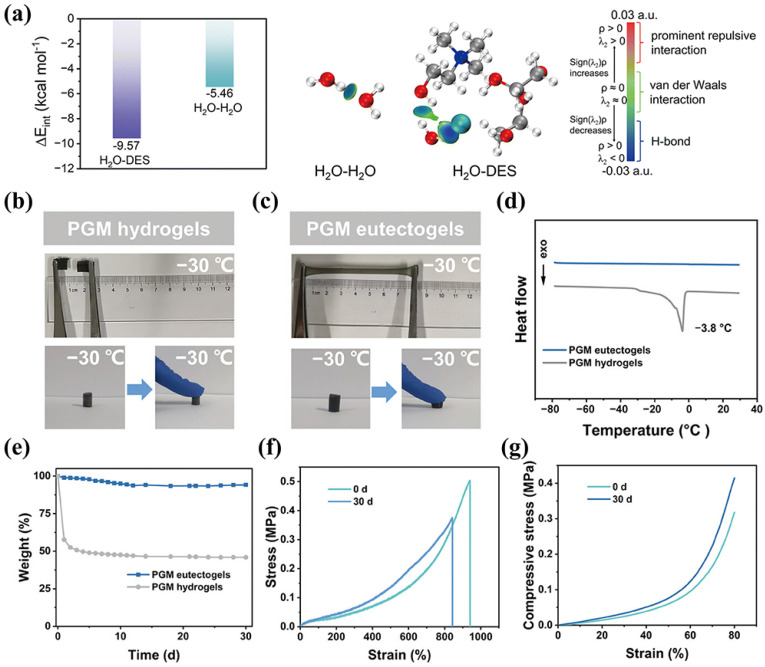
(**a**) Binding energy and independent gradient model based on the Hirshfeld partition (IGMH) three-dimensional diagram of H_2_O-H_2_O and H_2_O-DES, reproduced with permission from [71]. (**b**,**c**) Photographs of PGM hydrogels and PGM eutectogels stretching and pressing after freezing at −30 °C for 4 h. (**d**) DSC curves of PGM hydrogels and PGM eutectogels. (**e**) Weighs of PGM hydrogels and PGM eutectogels after storage for 30 days. (**f**) Tensile stress–strain curves and (**g**) Compressive stress-strain curves of PGM eutectogels before and after 30 days of storage, reproduced with permission from [34].

By forming a stable composite hydrogen bond network with a polymer matrix, the environmental stability of the eutectogel is significantly enhanced. Leveraging the comprehensive environmental stability provided by DES, such as wide temperature range, resistance to temperature and humidity fluctuations [68], anti-expansion, and long-term durability, the eutectogel has broken through the application boundaries of traditional gels. Depending on the characteristics of different types of DES, it can operate stably in extreme temperature environments [69], humid and underwater environments, dry and long-term use environments, alternating temperature and humidity, and complex media with mild acidity and alkalinity [70]. Moreover, the design of DES enables the customization of the environmental tolerance of the eutectogel, providing precise solutions for various practical applications and promoting the stable operation of eutectogel-based flexible devices in various complex environments. As shown in Figure 3a, DES exhibits a strong binding force, with H_2_O interfering with the hydrogen bonds between water molecules to form more stable associations [71]. This mechanism prevents the eutectogel from freezing at low temperature and losing water in dry environments. Yan et al. [72] introduced DES prepared from ZnCl_2_, ethylene glycol and glycerol into a eutectogel. Due to the synergistic effect of polyols and metal ions, this approach effectively overcame solvent freezing at low temperatures. The resulting eutectogel showed excellent low-temperature stability, maintaining good flexibility even after being stored at −20 °C for 1 month. Similarly, Guo et al. [34] developed PHEAA–gelatin–MXene (PGM) eutectogels which are constructed by poly(N-hydroxyethyl acrylamide)(PHEAA) and gelatin as their main components, with MXene nanosheets added as nanofillers, and with betaine and ethylene glycol as the DES. Compared with PGM hydrogel, the PGM eutectogel maintains good flexibility at low temperatures as shown in Figure 3b,c. As evidenced by DSC (Figure 3d), the eutectogel does not exhibit any phase transition behavior within the range of −80 °C to 40 °C. At the same time, the eutectogel can remain stable in the environment for 30 days and still possess excellent mechanical properties (Figure 3e–g). Liu et al. [73] have reported that eutectogel consisting of DES and MXene uniformly dispersed in poly(acrylic acid-acrylamide) (PAM) (MXene/DES@PAM) eutectogel, thanks to the anti-freezing property and high conductivity of DES which allow the eutectogel to maintain excellent ionic transport ability over a wide temperature range.

DES plays a pivotal role in the design of the environmental stability of eutectogels and is the cornerstone to their application in extreme environments. By tailoring the components of different DES, the functionalities of eutectogels can be customized for various harsh environments, thereby demonstrating their potential for practical application in a broader range of scenarios.

#### 2.1.3. Adhesion Properties

The ability of eutectogels to adhere effectively to the substrate is a prerequisite for their function as flexible sensors and detection devices, ensuring accurate electrical signal detection. Strong adhesion prevents slippage between the device and the substrate, thereby guaranteeing accurate signal acquisition.

**Figure 4:**
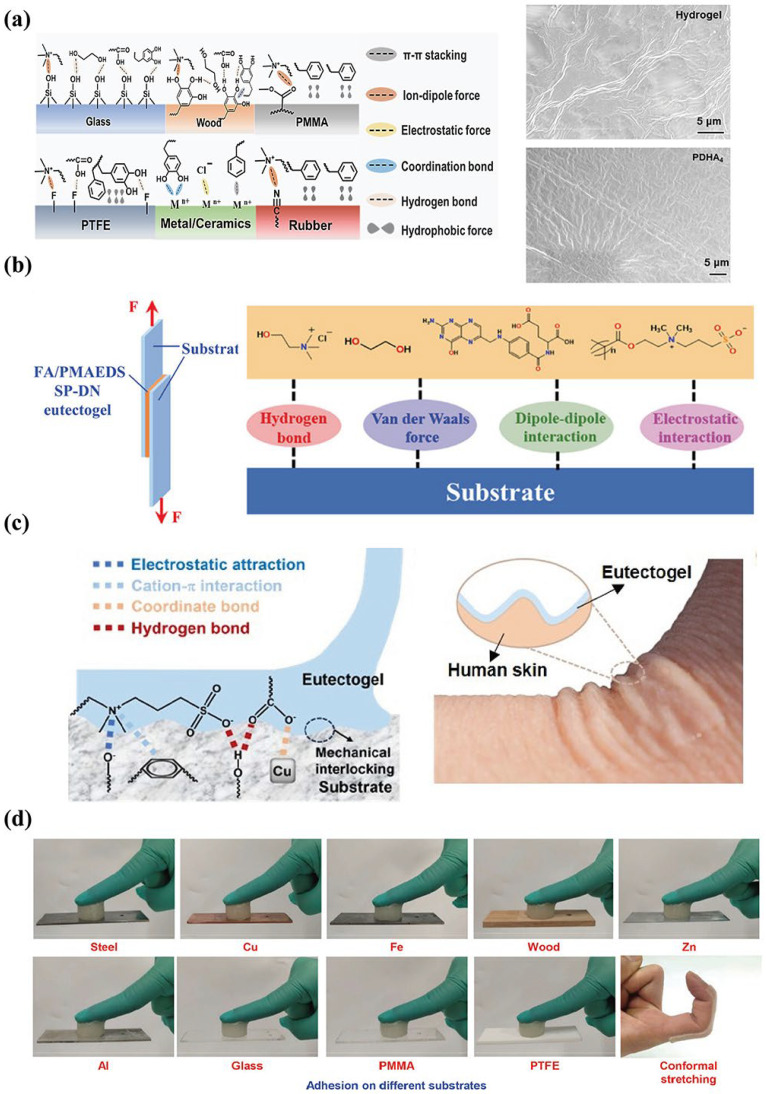
(**a**) Schematic illustration of possible adhesion mechanisms. Except for the mechanical interlocking effects, multiple weak forces emerge at the interface between eutectogels and substrates, reproduced with permission from [77]. (**b**) Schematic illustration of the lap-shear test geometry schematic diagram of the adhesion of eutectogel to different substrate materials, reproduced with permission from [79]. (**c**) Schematic of the adhesion mechanism between the eutectogel and substrates and photo showing eutectogel in full contact with skin, reproduced with permission from [29]. (**d**) Photographs of the eutectogels adhering to diverse substrates, reproduced with permission from [76].

The DES possesses abundant polar functional groups, such as hydroxyl, amino, amide, and carboxyl groups. It can form molecular-level non-covalent interactions such as interfacial hydrogen bonds [74], van der Waals forces [75], and electrostatic interactions [76] with the active groups of different substrate surfaces (such as metals, glass, polymers, and biological tissues), achieving robust bonding between the eutectogels and the substrate, significantly enhancing the interface bonding strength. DES can also form a composite hydrogen bond network with polymers, enabling the eutectogel to possess both moderate flexibility and cohesive strength. The gel can undergo conformal deformation in accordance with the rough structure of the substrate surface, creating a mechanical interlocking effect and firmly adhering to the substrate surface. As shown in Figure 4a, Jiang et al. [77] studied the adhesion behavior of mechanical interlocking effects and various non-covalent forces using acrylic acid (AA), dopamine methacrylamide (DMA), 2-hydroxy-3-phenoxypropyl acrylate (HPA), and 1,6-hexanediol diacrylate (HAD). The cured PDHA eutectogel and the hydrogel after water replacing DES verified their mechanical interlocking behavior and adhesion behavior under multiple interactions. SEM showed that after replacing the water, and when compared with the eutectogel, the hydrogel only showed irregular synapses, which hindered the full contact with the substrate, and the adhesion was weak. Moreover, Hou et al. [74] have demonstrated that eutectogels prepared from betaine and citrate DES adhered to tissues such as porcine skin and porcine bone via hydrogen bonds formed between the -NH_2_ and -COOH groups rich in the DES and those present on biological macromolecules, like polysaccharides and proteins. Similarly, Liu et al. [78] proposed four different adhesion mechanisms based on the adhesion of eutectogels to four different substrates. The functional groups within the eutectogels (hydroxyl, carboxyl, and amide) were found to form hydrogen bonds and ion–dipole interactions with Si–OH groups on glass substrates, hydrogen bonds and covalent crosslinks with metal ions, and anionic–π crosslinks with benzene rings on plastic surfaces. Consequently, the eutectogel tightly adhered to these diverse substrates, as shown in Figure 4b–d.

The rational selection of DES has a significant impact on the electrical conductivity, environmental stability and adhesion performance of the eutectogel. Among them, type III DES is the most commonly used in the preparation of eutectogel sensor devices. The robust hydrogen bond network formed between HBA and HBD significantly enhances the conductivity, environmental stability, and adhesion performance of the eutectogel. The abundant hydrogen bond groups possessed by DES interact with the matrix in multiple ways, enabling the eutectogel to firmly adhere to the measured objects, significantly reducing signal distortion and greatly improving the stability of the sensing. The multiple interpenetrating networks formed between DES and the copolymer matrix, as well as the excellent freeze resistance and anti-evaporation properties of DES, have greatly expanded the application scenarios of the eutectogel.

### 2.2. The Design of Eutectogel Networks

The preceding sections have already elaborated on the regulatory and supportive role of DES in controlling the properties of the eutectogel. The eutectogel is the core of flexible sensing devices, and it not only depends on the performance of the matrix network, but also lies in the integrated design of the surface and interface of the network structure. Rational structural design of eutectogels, such as their microstructure [80], can improve their sensing sensitivity. The dynamic network design can impart self-healing properties and high toughness to the sensing material, thereby effectively prolonging the service life of the device. Employing multi-network and composite strategies can enhance the mechanical strength, conductivity, and sensitivity of eutectogels.

#### 2.2.1. Microstructure Design

The introduction of microstructures to eutectogels improves the detection limit of gel-based sensing devices. The use of pyramids [81], microcones [82], and other structures [83,84], combined with advanced methods such as 3D printing, is expected to realize the detection of micro-deformation and micro-motion behavior.

**Figure 5:**
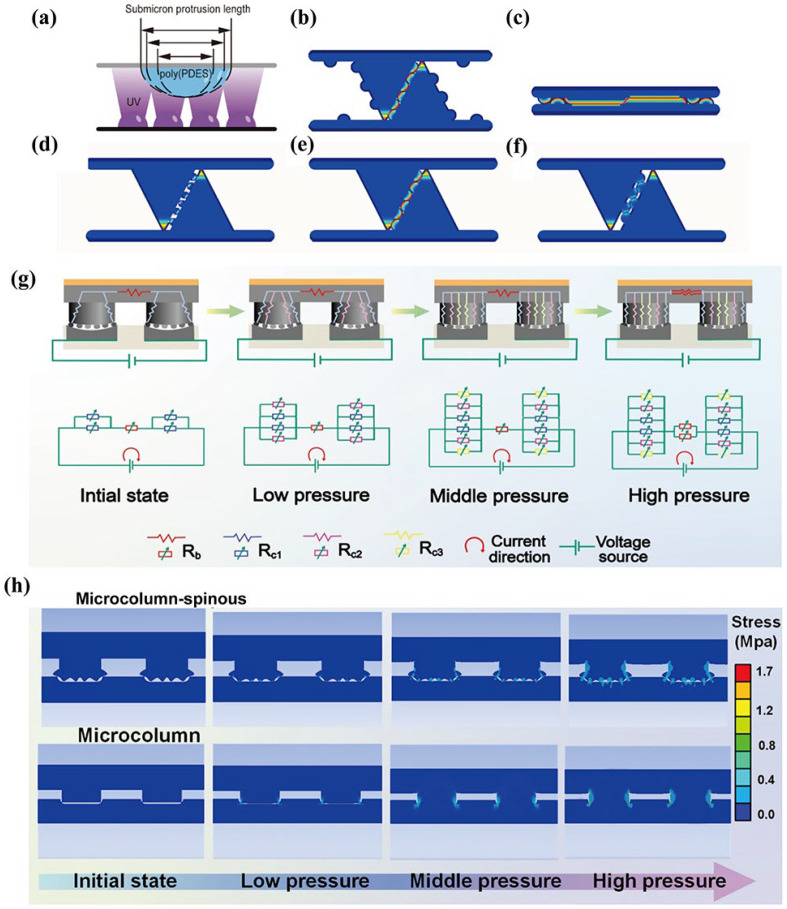
(**a**) Formation mechanism of submicrometer-scale protrusions on the surface of BIS during PDES polymerization, reproduced with permission from [88]. (**b**,**c**) Demonstration of the deformation of the unit structure when an external force acts on the bioinspired ion skin, reproduced with permission from [88]. (**d**–**f**) Finite element analysis of submicrometric-scale structures with different degrees of polymerized shrinkage in the sensing process, reproduced with permission from [88]. (**g**) Pressure-dependent evolution of conductive paths within the eutectogel/interdigital electrode tactile sensor under different pressure ranges and the corresponding equivalent circuit diagrams. (**h**) Finite element simulation of the local stress distribution on the contact surface, reproduced with permission from [39].

In contrast to sensors lacking microstructure, those incorporating microstructures generate huge local stress concentrations at the contact point or line under equivalent applied pressures [25], as shown in Figure 5a–f. Even a slight pressure is sufficient to induce significant elastic deformation at the tips of these microstructures [85]. This deformation will dramatically alter either the ion transfer path or contact resistance within the gel, thus amplifying the weak physical signal into a strong electrical signal. For weak physical signals, the deformation can be controlled by designing microstructures with different height, space, and stiffness. This effectively expands the linear response range of the sensor and avoids the saturation effect under high pressure.

Qiu et al. [39]. designed a multi-stage response microstructure eutectogel. From low contact pressure to high contact pressure, the hierarchical structure characterized by the multi-level contact mechanism leads to significant changes in the contact area during the whole compression process and the sensing performance is significantly improved, as shown in Figure 5g. As shown in Figure 5h, the design of the multistage structure is shown by using finite element analysis (FEA). Multistage structures have different contact behaviors under different pressures. For the unipolar structure, the pressure change is small and the sensitivity is low.

The eutectogel with microstructures enhances the structural deformation under external force stimulation by optimizing and monitoring the contact form at the interface, and strengthens the response feedback [86]. Meanwhile, by using the design of ordered arrays [87], the distribution of contact force is made more uniform, which alleviating the stress concentration and signal fluctuations caused by interface movement, thereby improving the stability and repeatability of the sensing signal. By directionally regulating the morphology, size and array density of the microstructures [26], it breaks through the limitation of mutually exclusive sensitivity and response range, broadens the dynamic response range, and realizes the continuous detection of weak signals to large deformation signals within the full range. However, these microstructures concurrently weaken the compressive strength of the eutectogel. Future research should therefore focus on developing new microstructures that exhibit improved mechanical and sensing properties, thereby expanding the range of detection of gel sensors.

#### 2.2.2. Dynamic Network Design

**Figure 6:**
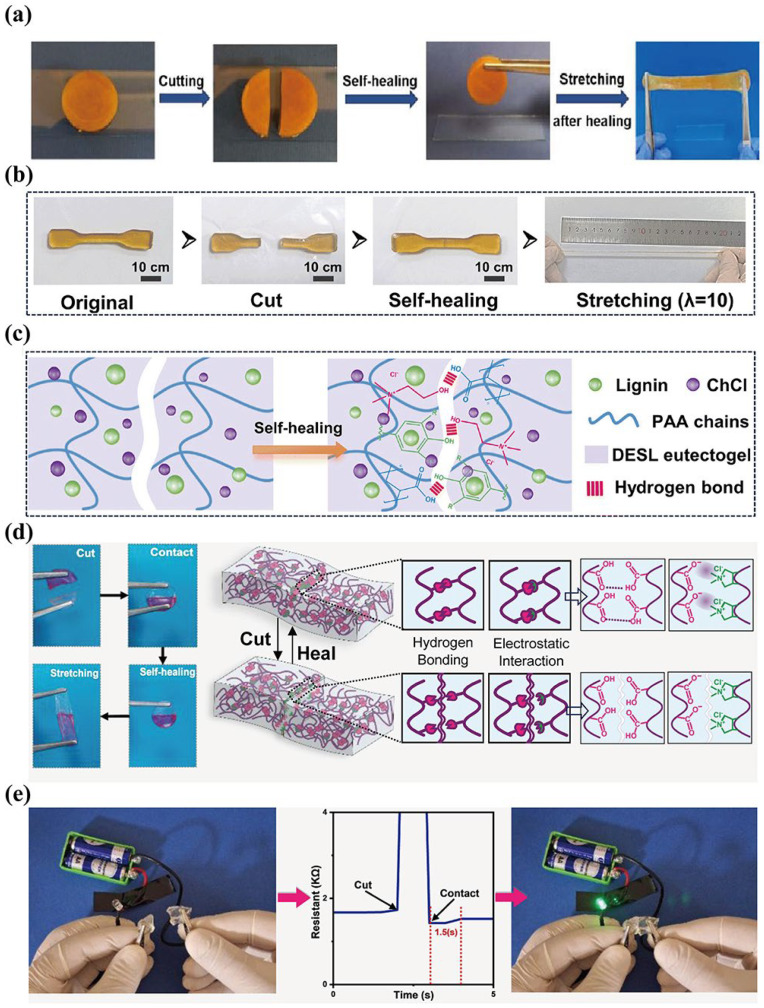
(**a**,**b**) Macroscopic investigation of eutectogel self-healing properties, reproduced with permission from [24,91]. (**c**,**d**) Schematic demonstration of the self-healing process of the eutectogel. (**e**) Digital images of the self-healing process of eutectogel in series with a green lamp, and its resistance graph showing that the eutectogel quickly returns to its original value, reproduced with permission from [24,41].

Owing to the existence of DES and the abundant hydrogen bonds, which serve as dynamic crosslinking groups within the eutectogels network, these materials exhibit excellent self-healing performance and toughness. When the eutectogels are damaged, the DES as a mobile phase rapidly fills the damaged area. At the interface, new hydrogen bonds rapidly form between the functional groups in the gel network and the DES components, as well as between the functional groups within the gel network itself [89]. The large number of hydrogen bonds between the HBA and HBD can promote the reconnection of the interface, functioning as molecular glue. Although the recovery efficiency does not reach 100%, the material’s performance can be restored to over 90% of its original level. Furthermore, other dynamic bonds can also be introduced into the eutectogels. For example, dynamic disulfide bonds [42] find it easy to break through the connection between sulfur atoms. However, the resulting sulfur radicals can reconnect to form an S–S covalent bond, or the electrostatic and covalent crosslinking can be established via a metal–ligand [90] coordination bond within the gel network, thereby achieving self-repair [91], as shown in Figure 6a,b. This self-healing effect is often attributable not to a single component but to the synergistic action of multiple functions. Liu et al. [24] and Yang et al. [4] used the eutectogel formed by choline chloride (ChCl) and acrylic acid (AA), and added phytic acid (PA) or lignin to the gel matrix. Through the breakage and recombination of the gel network and the additives, they achieved the strong self-healing property of the eutectogel. As shown in Figure 6d,e, researchers [41] developed an AA and gelatin eutectogel with diallyldimethylammonium chloride(DADMAC) and citric acid (CA). They cut the eutectogel to observe the self-repair behavior. During the repair process, the electrostatic bond and hydrogen bond between the AA functional group and DADMAC lead to the connection of damaged parts and the reconstruction of polymer network, so as to realize self-repair performance. Additionally, the eutectogel after self-repair still has excellent conductive behavior. In summary, the reasonable design of the dynamic network can significantly enhance the mechanical stability of eutectogels. This characteristic enables eutectogels to maintain a reliable sensing state in complex sensing scenes; even if the structure is damaged, the sensing performance can be rapidly recovered, which substantially reduces the operational cost of gel-based sensors. Therefore, the design of the dynamic network should be incorporated as a core element into the overall framework for optimizing eutectogel-based sensors’ performance.

#### 2.2.3. Multi Network and Composite Design

A single gel network struggles to simultaneously achieve high toughness, high conductivity, and stable sensing. By constructing multiple architectures and simultaneously compounding them with functional nanomaterials [7,92,93,94], the functions of different components can be coordinated, enabling the fabrication of eutectogel-based sensor devices with superior performance.

Although highly crosslinked single-network eutectogels exhibit high strength, the soft single-network structures are prone to creep due to their low sensitivity, resulting in poor signal stability [95]. Therefore, a multi-network composite strategy is adopted to assign specific functions to the most suitable components, achieving synergistic performance improvements. For instance, Shan et al. [96] designed a eutectogel using polyacrylamide (PAM), gelatin, and quaternized chitosan, and incorporated the conductive polymer poly(benzodifurandione) (PBFDO). Gelatin and quaternized chitosan introduced a large number of hydrogen bonds, endowing the eutectogel with excellent mechanical properties. The addition of PBFDO improved the sensing performance of the eutectogel, yielding a gauge factor (GF) of 3.03 and a conductivity of 0.47 S/m. Similarly, Chang et al. [43] constructed a core–shell composite structure using polyvinyl alcohol (PVA), sodium alginate (SA), and poly(3,4-ethylenedioxythiophene)-poly(styrenesulfonate) (PEDOT: PSS). The eutectogels developed by this method showed capabilities for wide-range strain detection and temperature sensing, fully leveraging the advantages of each component of composite materials.

The multi-network synergy and composite design takes the eutectogel network as the core. By leveraging the structural complementarity, performance synergy, and functional diversity of different networks [97], it compensates for the performance limitations of a single eutectogel network and broadens the functional boundaries. By interpenetrating the highly cross-linked network and the low-cross-linking dynamic network [98], the problem of the inability to achieve both flexibility and strength, as well as fatigue resistance and structural stability in a single network, is solved. The introduction of specific stimulus-responsive monomers [99,100] into the eutectogel enables it to upgrade from a single-stimulus signal conversion to a multi-functional integrated sensing system.

To sum up, the design of the sensing performance of eutectogels lies in the coordination between the reasonable selection of DES and the fine design of the gel network. As the continuous phase of the gel, the type of DES directly influences the conductivity and environmental stability of materials. Among these, Type III DES has emerged as the predominant choice due to its wide selection range of HBD and HBA, which can not only allow for the adjustment of conductivity as required but also enables the gel to adapt to a wide temperature range environment through specific component selection. Through microstructure design, the detection accuracy and the response range are enhanced. With the help of dynamic network design, self-healing capability and high toughness are realized, which effectively extends the service life. Furthermore, a multi-network composite strategy can be employed to optimize both mechanical strength and sensing sensitivity. The synergistic effect of these principles overcomes the limitations of traditional gels, providing a fundamental basis for high-performance sensing applications.

## 3. Sensing Mechanism of Eutectogels

At present, the sensing mechanism based on eutectogels mainly centers on three major paths: resistance [101], capacitance [102], and triboelectric [103]. Each mechanism, operating on distinct physical principles, converts external mechanical or environmental stimuli into electrical signals, thereby achieving the sensitive monitoring of multimodal information such as deformation, pressure, touch, and motion. These three mechanisms complement each other, collectively expanding the application potential of eutectogel in fields including wearable electronics [104], health monitoring [1], and human–computer interaction [105], promoting the development of flexible sensing technology toward multi-function, adaptive, and high integration.

### 3.1. Resistive Sensing

**Figure 7:**
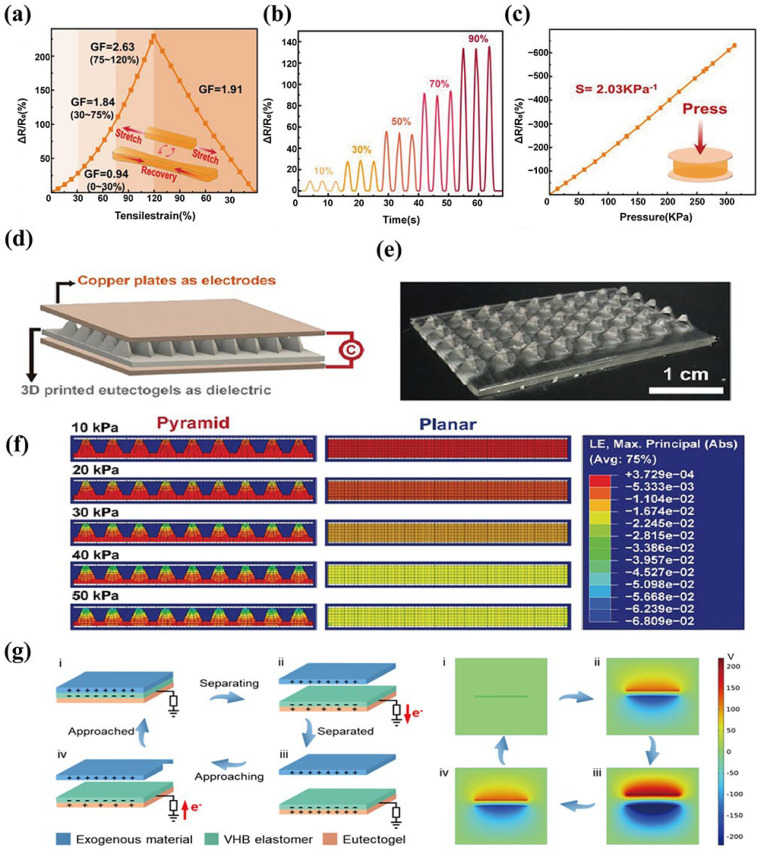
Resistance response under different (**a**,**b**) tensile strains and (**c**) compressive strains, reproduced with permission from [116]. (**d**) Design of the 3D-printed capacitive sensor, reproduced with permission from [108]. (**e**) Photo of the 3D-printed pyramid dielectric, reproduced with permission from [108]. (**f**) Finite element analysis of the pyramid and planar dielectric under different pressures, reproduced with permission from [108]. (**g**) Schematic diagram of the eutectogel triboelectric sensor and Finite Element Analysis of potential distribution, reproduced with permission from [117].

Resistance sensing is the most common and direct sensing mechanism in eutectogels. Without external force, internal ions migrate in the gel network, forming a stable ionic conductive path [105]. The resistance change is usually expressed as R=ρL/A. When the gel is subjected to an external force, the three-dimensional network structure inside the gel undergoes deformation, and the newly formed ion transport channel becomes longer and more complex, leading to the resistance change. As shown in Figure 7a–c, under different tensile strains and compressive strains, the change rate of resistance is also different. Compared with low strains, high strains result in a longer ion transmission distance and a more significant change in resistance, which realizes the identification of different strains.

Moreover, the dynamic composite hydrogen bond network of eutectogel possesses a reversible dissociation-recombination capability. After the external stimulus is removed, the hydrogen bonds recombine rapidly, the network structure will return to the initial state, and the ion migration behavior will be restored simultaneously, and the gel resistance will also return to the initial value without obvious signal hysteresis and residue. This feature enables the eutectogel resistive sensor to adapt to the repeated dynamic mechanical deformation in flexible sensing (such as the flexion and extension of human joints and the continuous vibration of equipment), ensuring the repeatability and accuracy of the sensing signal.

The ionic conductive intrinsic property of resistive sensors can be transformed into the core advantage for temperature detection. The ion carriers dissociated from DES in the eutectogel will undergo significant and reversible regulation in terms of their migration rate, activity and migration resistance with changes in temperature [106]. This induces a stable and quantifiable linear change in the gel matrix resistance, enabling the high-sensitivity capture of temperature signals without the need for complex modifications.

### 3.2. Capacitive Sensing

Capacitive sensors typically comprise a conductive layer and a dielectric layer, as shown in Figure 7d. Eutectogels serve as the dielectric layer. Under external stimulation, the eutectogels deform, inducing structural changes in the structure of the dielectric layer. Consequently, the capacitance of the sensor device changes, enabling the conversion of external physical signals into electrical signal outputs [107]. This working principle can be described by the basic formula of the parallel plate capacitor. $C=\varepsilon_{0}\varepsilon_{r}A/d$, where C is the capacitance, $\varepsilon_{r}$ represents the relative dielectric constant of the dielectric layer, A is the effective overlapping area between conductive plates, and d is the thickness of the dielectric layer. When pressure is applied, compression of the eutectogel dielectric layer causes d to decrease, thereby increasing the capacitance.

Sheng et al. [108] synthesized capacitive sensors based on menthol and decanoic acid-based acrylic acid and fabricated the eutectogel dielectric layer with a pyramid structure through 3D printing technology (Figure 7e). As shown in Figure 7f, Finite Element Analysis was used to demonstrate the deformation process of the pyramid dielectric layer under different pressures, achieving accurate identification under these different pressures. Compared with the dielectric layer without microstructure, its pressure sensing capability was significantly improved. Liu et al. [109] fabricated a eutectogel-based capacitive sensor that achieved temperature-dependent sensing by leveraging the influence of temperature on the ion migration within the eutectogels. Meanwhile, they used simulations to visually demonstrate the process of capacitance change in the gel under different pressures.

### 3.3. Triboelectric Sensing

In the eutectogel flexible sensing device, a self-powered device based on triboelectric nanogenerator (TENG) technology has also been widely studied. The triboelectric effect is a kind of mechanical stimulation that can be converted into electrical energy and which can supply power to small equipment. The fundamental principle of the triboelectric effect is based on the coupling effect of contact electrification and electrostatic induction [110]. As shown in Figure 7g, when the surfaces of two materials with distinct dielectric properties contact, charge transfer occurs at the interface due to the difference of their electronic gain and loss ability. When connected to an external circuit, the induced current can power small equipment, breaking the dependence of traditional sensors on external power supplies.

Among the four contact electrification modes of TENG, the eutectogels triboelectric sensing devices commonly use single electrode [111] and contact-separation modes [112]. Eutectogels can not only be used as a friction dielectric layer to generate electrical signals through interface transfer, but also as a conductive electrode to transfer charge. Chu et al. [113] developed electronic skin based on the triboelectric principle utilizing a metal-based DES. By using the contact-separation mode, they further verified the output behavior of eutectogel-based TENG under different contact frequencies and different contact areas. With the increase of contact frequency, the open-circuit voltage basically remained unchanged, and the short-circuit current increased with the increase of frequency. By varying the contact-separation pressure, frequency, area, etc., the electrical signal output is realized.

The intrinsic properties of the eutectogel directly determine the electrical signal output performance during the electrostatic induction stage by regulating the charge transfer, capture retention and interface contact efficiency of the triboelectric effect. The high polarity of DES in the eutectogel can modulate the surface work function of the gel [114], creating a larger work function difference with the heterogeneous friction layer, providing a larger charge base for electrostatic induction.

The resistance type eutectogel flexible sensor device directly uses the eutectogel as a sensing unit without complex electrode design. Moreover, it can directly output resistance signals. The capacitive eutectogel sensor has a linear relationship between capacitance and deformation pressure, which can directly decouple the mathematical expression between capacitance and pressure. The dielectric layer can introduce microstructure to realize a generational improvement in sensing sensitivity. The eutectogel sensor of the triboelectric nanogenerator effectively addresses the aforementioned two drawbacks that limit applicability in passive scenarios, and can convert mechanical signals into electrical signals. At the same time, it integrates the advantages of TENG and eutectogels, enabling it to achieve stable triboelectric response in extreme environments; however, during the long-term contact separation processes, the surface of the eutectogel is prone to wear [115], which leads to signal attenuation.

## 4. Eutectogels for Sensing Applications

Performance is the foundation of applications, and applications are the ultimate interpretation of performance. The high ionic conductivity and wide potential window provide the basis for constructing high-sensitivity sensors. This section will focus on the sensing application of eutectogel as a core sensitive material or functional matrix, and will systematically describe how its unique material properties can be transformed into innovative sensing solutions. In Table 3, the composition of eutectogels, the performance parameters of the sensing mechanism, and their applications are systematically summarized.

### 4.1. Flexible Strain/Motion Sensing

Eutectogel, as a potential substitute for hydrogels, has been widely used in the field of flexible sensing. The deformation of eutectogel changes its internal conductive network and thus produces measurable electrical signal changes [125].

**Figure 8:**
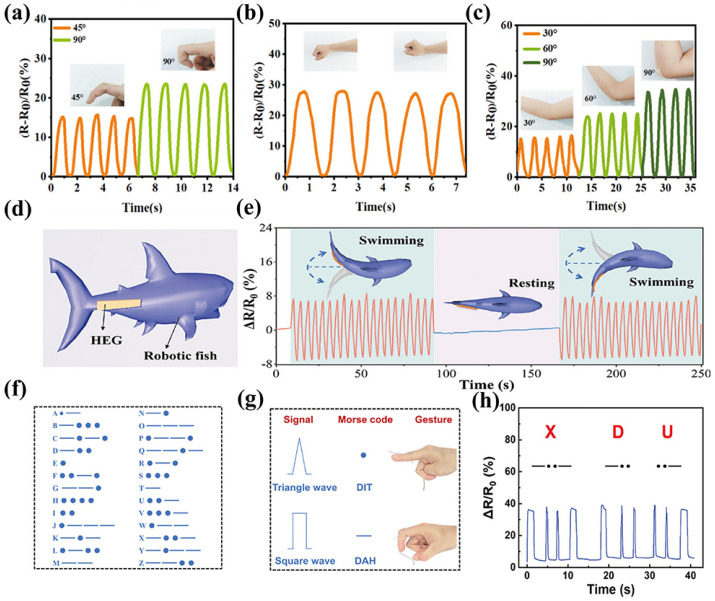
The eutectogel strain sensor for monitoring human motions: (**a**) Finger bending, (**b**) Wrist bending, (**c**) Elbow bending. (**d**) Schematic illustration of the sensor attached to the right side of the robotic fish model. (**e**) The relative resistance variations when the robotic fish model is swimming and resting, reproduced with permission from [127]. (**f**) Morse code table for 26 letters. (**g**) Schematic diagram of signal codes. (**h**) Electrical signals corresponding to “XDU”, reproduced with permission from [129].

As shown in Figure 8a–c, eutectogels can adhere to the skin and capture the real-time bending amplitude and frequency of joints such as fingers, wrists, and the elbow, realizing joint motion sensing. Meanwhile, due to the large number of hydrogen bonds introduced by DES, a stable energy dissipation mechanism is constructed, one which endows the eutectogels with excellent fatigue resistance and ensures the long-term service stability of eutectogel-based sensors and monitoring devices [44].

In order to enable eutectogels to meet the strain sensing requirements under multiple scenarios, Hao et al. [126] synthetized eutectogels of acrylic acid and 2,2,2-trifluoroethyl methacrylate through the use of chlorocitrate ethylene glycol (molar ratio: 1:2) DES and by employing a photoinitiated polymerization reaction, which forms a hydrophilic/hydrophobic heterogeneous network. After immersion in salt, alkali, acid, and organic solutions for one month, the eutectogels maintained good mechanical properties (tensile strength: 90 kPa) and electrical conductivity (70 mS/m, 70% of original value). This excellent solvent resistance enables it to achieve stable sensing underwater. As shown in (Figure 8d,e), researchers [127] prepared a hydrophobic DES composed of tetrabutylammonium tetrafluoroborate and succinonitrile (in a 1:4 ratio), and used it to synthesize acrylate eutectogel. The eutectogel demonstrated excellent underwater detection capabilities and they attached the eutectogel to the right side of the robotic fish model in order to monitor the movement. Eutectogel can well distinguish between swimming and resting state through changes in ΔR/R_0_. Raffa et al. [128] employed the same approach by integrating soft hydrophobic domains (i.e., butyl acrylate, BA), thereby realizing strong adhesion to various substrates and reliable sensing performance. As shown in Figure 8f–h, Ding et al. synthesized ChCl and glycerol (molar ratio 1:2) DES through heating and stirring, and then formed a eutectogel by combining PEDOT: PSS, gelatin and borax. Subsequently, they compounded it with thermoplastic polyurethane (TPU) to obtain the synthesized composite gel, which exhibited excellent electrical conductivity (0.06 S/m) and sensing capability (GF = 2.52, 600% strain). Additionally, they used the dots and dashes in the Morse code through different finger movements. By using different codes and the sequence of movements, they were able to accurately recognize and output the letters “X”, “D”, and “U”. The eutectogel resistive sensor also provides a paradigm for secure communication.

Eutectogel flexible strain sensing converts stretching, bending and other strain stimulation into resistance signals, achieving resistance response through the change of ion migration pathways. The eutectogel resistive sensor is designed based on DES and a gel network and it exhibits advantages such as high flexibility and environmental stability (including anti-swelling property, heat resistance, acid and alkali resistance, etc.), meaning that is widely applied in wearable motion detection and deformation sensing fields.

### 4.2. Microscopic Deformation Sensing

Across sensing applications involving different deformation scales, eutectogels are required to meet distinct performance requirements. For sensing under large-deformation conditions, robust sensing performance must be guaranteed during the loading and unloading of external forces. In contrast, for the detection of subtle deformations caused by movements such as facial expressions and swallowing, eutectogels require highly sensitive sensing capabilities.

**Figure 9:**
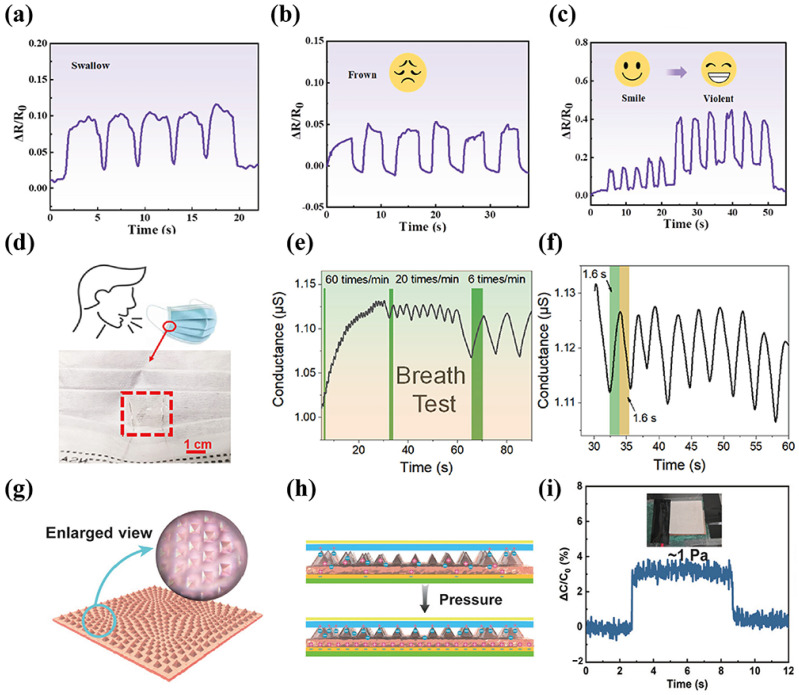
Application of eutectogel sensor in detecting human motion: (**a**) swallow, (**b**) frown, and (**c**) smiling and laughing, reproduced with permission from [130]. (**d**) Schematic diagram of the breath test with eutectogel sensor adhered to the face mask. (**e**) Conductance response curves at different respiratory rates of 60 times/min, 20 times/min and 6 times/min. (**f**) The response and recovery time in respiratory monitoring cycles with the rate of 20 times/min, reproduced with permission from [120]. (**g**) Schematic diagram of pressure detection of micro-structure hydrogel. (**h**,**i**) Minimum 1 Pa pressure detection capability, reproduced with permission from [121].

Zhao et al. [53] synthesized eutectogels by in situ polymerization of poly(3,4-ethylenedioxythiophene) (PEDOT) within carboxymethyl cellulose (CMC), and through synergistic interaction with DES. The obtained eutectogels demonstrated high stability and sensitivity. This enables the eutectogel to effectively detect microdeformations associated with swallowing, frowning, smiling and other physiological signals (Figure 9a,b). Similarly, Che et al. [119] used a ternary DES (ChCl:AA:Urea = 1:2:2) and combined it with PVA to synthesize a eutectogel through photoinitiated polymerization. Thanks to the inherent ionic conductivity of DES, the eutectogel exhibited excellent sensitivity over a wide range of strains (gauge factors (GFs) of 1.95, 3.12, and 3.30 for strain ranges of 0–30%, 30–70%, and 70–100%, respectively), enabling it to have excellent detection capabilities for both large deformations and small deformations. They utilized the eutectogels for vocal cord vibration sensing and successfully distinguished between the two words “Hydrogel” and “Sense”, with corresponding relative resistance changes of 16.12% and 3.13%, respectively. This demonstrates its outstanding sensitivity toward subtle physiological signals. Li et al. [120] synthesized a eutectogel by combining gelatin with DES (with a sorbitol to citric acid molar ratio of 2:3). Leveraging the abundant polar groups of gelatin and DES to capture moisture from the environment, the absorption of water molecules reduces its viscosity and activates the ionic transport pathways within the gel network, thereby enhancing conductivity. This results in an accurate correspondence between respiratory frequency and electrical signals. Additionally, they achieved the measurement of breathing frequency by attaching eutectogel to the inner side of a mask, taking advantage of its special response behavior during breathing. The eutectogel can accurately distinguish breathing frequencies of 60 times/min, 20 times/min, and 6 times/min, and has a response time of 1.6 s, as shown in Figure 9d–f.

To address the need for enhanced microdeformation sensing performance in eutectogels, the scope for performance improvement is severely restricted by merely adopting physical mixing or chemical doping of conductive materials. In contrast, the design and introduction of specific gel microstructures has emerged as a critical and effective strategy to break through the limitations of eutectogels in terms of sensing sensitivity. Feng et al. [121] combined the gold nanoparticles synthesized in the ternary DES (ChCl/EG/Urea) with acrylic acid and polyvinylpyrrolidone, and then used the template transfer method to synthesize a eutectogel with multi-level pyramid microstructures, as shown in Figure 9g,h. The triangular pyramids in each quadrant diminish gradually from the circumference to the center, enabling a pressure response at a pressure as low as 1 Pa (Figure 9i).

The incorporation of conductive materials and microstructural design can effectively strengthen the capability of eutectogels for microdeformation recognition, thus extending its application range in microdeformation sensing. Nevertheless, it is important to emphasize that in micro-event sensing scenarios, the output signal strength of such gels remains relatively weak. Consequently, achieving a stable output of high-fidelity signals has become a primary focus for subsequent research regarding the enabling of eutectogels to accurately identify subtle changes.

### 4.3. Electromyogram (EMG) and Electrocardiogram (ECG) Sensing

Eutectogels have been successfully constructed as a universal and robust flexible sensing platform from macro joint movement to micro physiological vibration.

As for the EMG and ECG signals with more complex scenes, because the signal is vulnerable to movement interference, sweat corrosion leads to performance degradation, poor long-term wear comfort and other problems. Given these issues, it is difficult for traditional hydrogels to achieve high-accuracy signal output. The detection of EMG and ECG originates from the periodic and micro-scale deformations and micro-pressure fluctuations of the body surface skin caused by the contraction and relaxation of skeletal muscles [131]. By applying the eutectogel sensor to the EMG collection area, its low modulus and high adhesion properties enable a seamless and tight contact with the skin. The micro-deformations of the skin will be simultaneously transmitted to the gel interior, triggering small expansions and densifications of the dynamic hydrogen bond [132] network [133], which directly alters the migration path, rate, and carrier distribution of the DES dissociated ions, causing the resistivity of the gel body to exhibit synchronous and periodic reversible fluctuations in response to the micro-deformations of the skin. Eventually, the resistivity fluctuations of the gel will be converted into corresponding voltage or current electrical signals by the sensing system. By collecting the amplitude, frequency, and waveform characteristics of these signals, the physiological features of the EMG can be restored, and the detection of the electromyographic signal can be completed.

**Figure 10:**
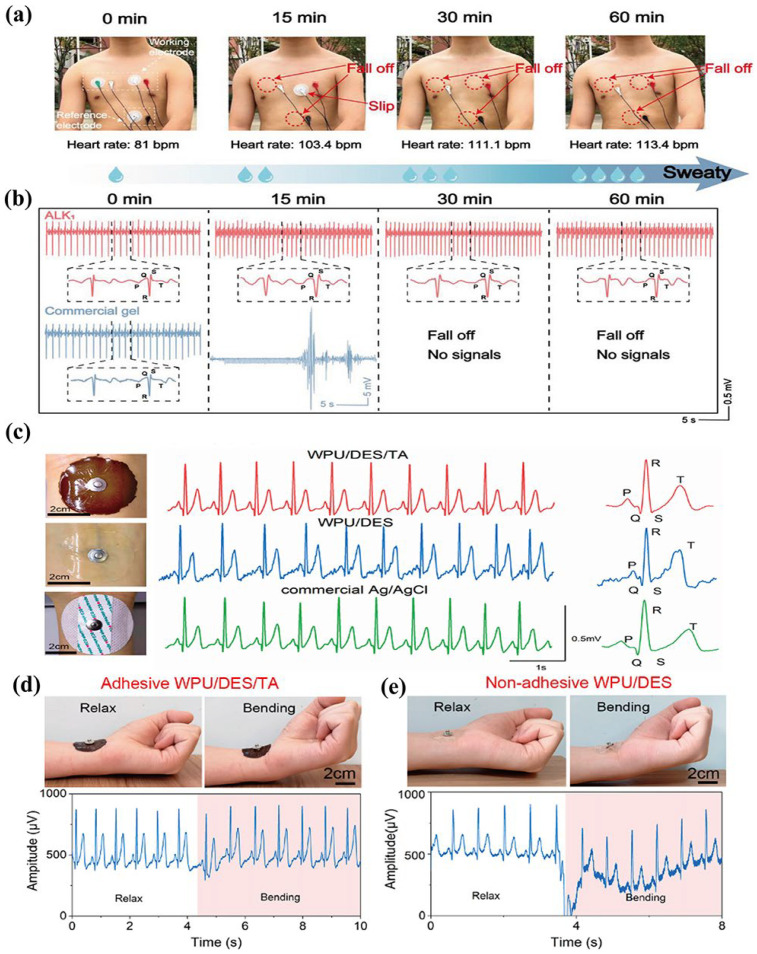
(**a**) Photos of ALK eutectogel and commercial gel electrodes adhered to human skin. (**b**) The corresponding ECG signals recorded by ALK eutectogel and commercial gel electrodes under different exercise times, reproduced with permission from [122]. (**c**) Comparison of the ECG signals using commercial Ag/AgCl, adhesive eutectogel and non-adhesive eutectogel electrodes. ECG signals recorded with (**d**) self-adhesive eutectogel and (**e**) non-adhesive eutectogel electrodes during the wrist bending, reproduced with permission from [1].

Li et al. [122] prepared a fully physically crosslinked eutectogel. The multiple hydrogen bond interactions enable the eutectogel to achieve seamless adhesion to the skin, even under sweating conditions. Meanwhile, it also exhibits excellent biocompatibility and low interfacial contact impedance characteristics. As a skin electrode, it has an excellent anti-interference ability against motion artifacts and sweat. As shown in Figure 10a,b, commercial electrodes failed after 15 min of movement, while the synthesized eutectogel electrode prepared by DES, AA, lignin and konjac glucomannan (the obtained eutectogel called ALK) can still accurately detect the ECG signal in the case of massive sweating in 60 min. Additionally, the impedance of ALK (235.8 kΩ at 1 Hz) is significantly lower than the commercial gel (368.6 kΩ at 1 Hz) in the frequency range of electrophysiological signal acquisition under normal conditions. This ensures that the eutectogel can accurately and promptly convey physiological health information. Wang et al. [1] prepared DES using ChCl and glycerol (molar ratio: 1:2), which was then mixed with waterborne polyurethane (WPU) and tannic acid (TA). Due to the presence of DES, the eutectogel electrode exhibits excellent environmental stability (it can maintain good flexibility even at −20 °C) and outstanding EMG detection capability, as shown in Figure 10c. Moreover, thanks to the addition of polyphenol TA, the eutectogel can better adhere to the skin surface. Compared with the eutectogel without TA added, it has stronger adhesion (the peel strength is 10 times the original value (3 N/m)) and a more stable output (Figure 10d,e).

By virtue of its unique and excellent comprehensive properties, eutectogels provide a powerful material solution to addressing the challenge of long-term, stable, and high-fidelity EMG signal monitoring. With the continuous innovation in material design and the constant expansion of application scenarios, eutectogels are expected to drive flexible wearable electronic technology to achieve new breakthroughs in fields such as medical rehabilitation, sports science, human–computer interaction, and even special environment monitoring [134].

### 4.4. Temperature Sensing

**Figure 11:**
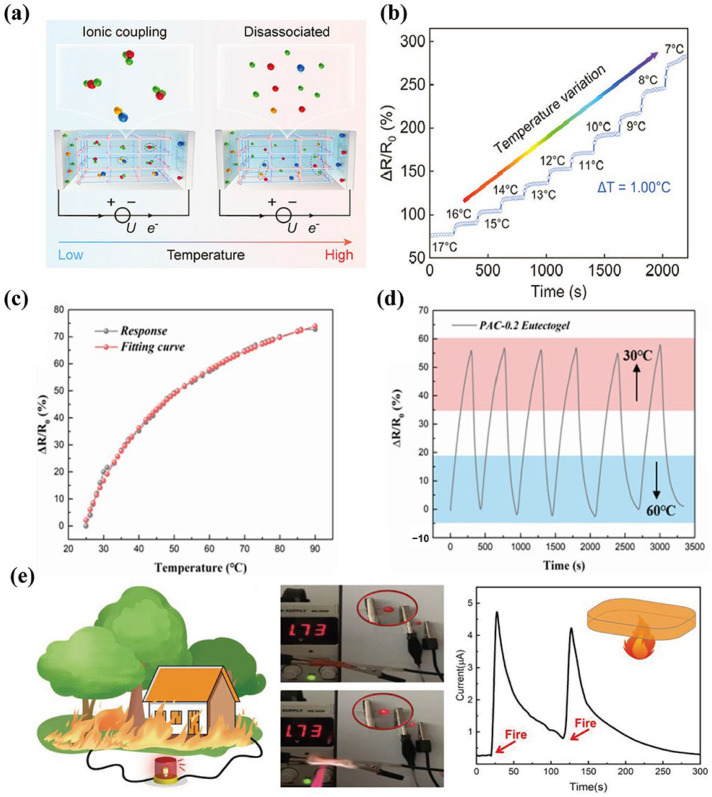
(**a**) Schematic of the ion coordination dynamics mechanism. (**b**) High-resolution detection of 1.0 °C variations, reproduced with permission from [45]. (**c**) Temperature sensitivity and (**d**) temperature-sensing cycle stability of eutectogels temperature sensor, reproduced with permission from [136]. (**e**) Schematic diagram of early warning of house and forest fire, reproduced with permission from [116].

Without the addition of conductive materials, the electrical conductivity of eutectogels is primarily dependent on the migration of free ions within DES, whereas temperature has a notable impact on the ion migration rate of DES [135]. As the temperature rises, the viscosity of DES decreases, leading to a reduction in the movement resistance of ions in the eutectogels. Meanwhile, heat supply provides more energy to the ions and accelerates their movement rate. The synergistic effect of these two factors jointly leads to a significant increase in the ionic conductivity of the gel with increasing temperature, which is macroscopically manifested as a decrease in resistance. Therefore, eutectogels can be used as temperature sensors. As shown in Figure 11a,b, the eutectogel realizes the temperature sensing behavior at 1 °C change.

Zhu et al. [45] formed P(NIPAM-co-VA)/LiTFSI/ZnCl_2_ eutectogels by a coordination-drive molecular strategy, in which N-isopropylacrylamide (NIPAM) and vinyl acetate (VA) form the copolymer network and lithium bis(trifluoromethanesulfonyl)imide (LiTFSI)) and ZnCl_2_ are used as ionic ligands. The designed dynamic coordination network confers the gel with outstanding thermosensitivity and intrinsic mechanical softness. The obtained eutectogel sensor exhibited an ultra-high temperature resolution of 0.05 °C, enabling real-time monitoring and early detection of abnormal temperature fluctuations in cold chain transportation. Wang et al. [136] correlated the temperature-dependent diffusion behavior of chloride ions and choline cations in DES (ChCl:EG = 1:2) with temperature, demonstrating the sensing performance of the eutectogel with stable temperature under thermal cycling (Figure 11c,d). Ge et al. [123] synthesized a glycerol–AA-based eutectogel and combined it with polyaniline nanofibers (PANI NFs). The thermosensitive trait of PANI NFs also endows the strain sensor with superb TCR (183.71 °C^−1^) and high temperature resolution of 2.7 °C, meaning that it could be applied in fire alarm devices. As shown in Figure 11e, upon exposure to flame, the LED brightness increased gradually within seconds, exhibiting the eutectogels capability to detect high-temperature flames and activate an alarm. When the flame was extinguished, the eutectogel combustion ceased abruptly, displaying self-extinguishing behavior. The eutectogel combines the low volatility and low freezing point characteristics of DES, rendering the temperature response range wider (−20–90 °C). Moreover, the reversibility of the dynamic network ensures that the sensing signal does not have significant baseline drift during temperature cycling changes, and has good repeatability. It also shows anti-expansion and anti-drying environmental tolerances and does not require additional moisturizing protection. It can stably measure temperature in various complex working conditions. These functional materials with unique advantages have gradually broken through the limitation of basic laboratory research and expanded to multiple engineering and practical fields, like industrial temperature monitoring, showing their great potential and core value in practical applications.

### 4.5. Extreme Environment Sensing

**Figure 12:**
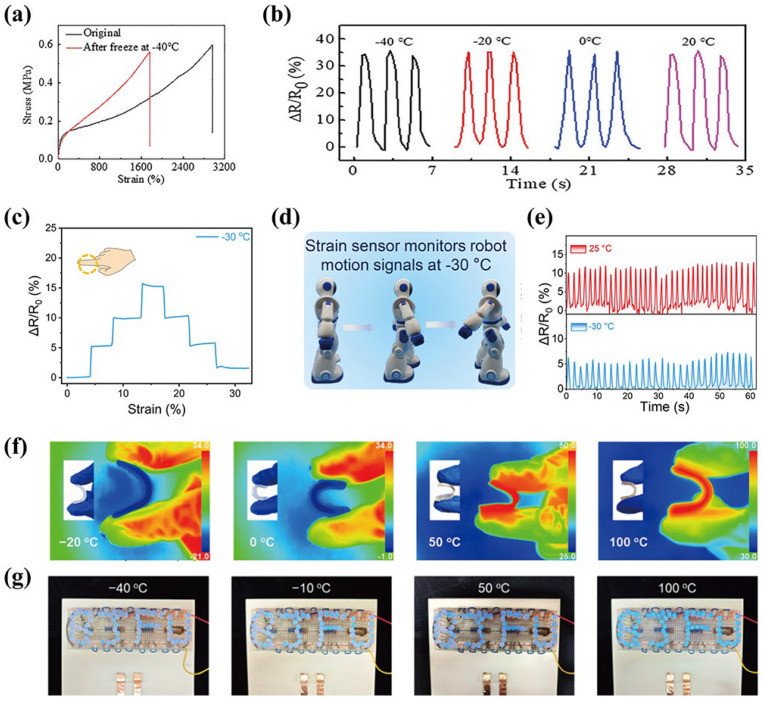
(**a**,**b**) Relative resistance changes by finger bending at different temperatures, reproduced with permission from [125]. (**c**) The sensor monitors the resistance signal change of finger bending at 30°, 60°, and 90° at −30 °C. (**d**,**e**) The sensor is attached to the robot’s knee to monitor the motion signal during walking, reproduced with permission from [63]. (**f**) Thermal images of eutectogel bending at approximately −20, 0, 50, and 100 °C. Insets: the corresponding optical images. (**g**) Photographs of eutectogel acting as conductor to light up LEDs at different temperatures, reproduced with permission from [117].

Eutectogels have excellent anti-freezing and drying resistance properties, which makes them break through the performance bottleneck of traditional sensing materials under extreme temperature and humidity conditions, endowing them with stable operation over a wide temperature range. Even in harsh environments such as low-temperature freezing or high-temperature drying, their sensing signal can remain accurate and stable. For example, Zhang et al. [124] developed a liquid metal-doped choline chloride–ethylene glycol (ChCl-EG)-based eutectogel that maintains high flexibility after storage at −20 °C for 7 days, demonstrating outstanding electrical conductivity over a temperature range of −20 °C to 50 °C. Sun et al. [137] fabricated an autocatalytic system of eutectogels utilizing the solvent replacement method, enabling rapid gelation within 30 s. As shown in Figure 12a, the eutectogel still maintains excellent mechanical properties even at −40 °C. Compared with hydrogels, the eutectogels possess the ability to achieve good sensing performance over a wide temperature range (−40–20 °C) as shown in Figure 12b. Even after being damaged artificially, their abundant dynamic groups can quickly repair the damaged areas and maintain the original output performance almost unchanged. Ren et al. [63] studied the chlorocitrate ethylene–glycol-based PVA and PAA eutectogel. DES gave the eutectogel low-temperature freeze resistance, enabling the gel to have excellent detection capabilities at −30 °C. At the same time, the eutectogel was installed in the robot’s knee area and placed in a −30 °C environment to monitor the signals generated when the knee bends during walking. The bending signal showed that the conditions at 25 °C and −30 °C were highly similar, and the sensor exhibited excellent stability and repeatability at low temperatures (Figure 12c–e). Sun et al. [117] utilized the inherent low freezing point and vapor pressure of lactic acid(LA)/ChCl DES to achieve a significant anti-freezing performance. The eutectogel exhibited remarkable flexibility within the temperature range of −20 °C to 100 °C (Figure 12f). Additionally, as a conductor, it could light up multiple small bulbs at different temperatures as shown in Figure 12g, demonstrating its sensing performance in extreme temperature environments.

In the fields of low-temperature sensing, eutectogels have achieved breakthroughs from material innovation to application [30]. However, they still face challenges such as synergistic improvement of sensitivity and self-healing efficiency, low-temperature interfacial stability optimization, and large-scale preparation under low-temperature conditions. In the future, multifunctional integration, intelligent response design, and enhanced environmental adaptability will be emphasized to expand their application boundaries in extreme environments such as polar scientific research and deep space exploration [138].

## 5. Conclusions and Outlook

As a new type of flexible functional material with DES as the continuous phase, eutectogels successfully overcome the inherent limitations of traditional hydrogels and organogels in terms of susceptibility to freezing, volatilization, and leakage, thereby realizing synergistic improvement in both environmental stability and comprehensive sensing performance. This review systematically summarizes the research framework of eutectogels from the selection of DES, the optimization of the gel network structure, the sensing mechanism, and a variety of applications. The findings show that the mechanical properties, environmental adaptability, and functional integration of eutectogels can be improved through reasonable DES selection and refined gel network design. This lays the theoretical foundation for the design of high-performance eutectogel-based sensing and monitoring devices.

To sum up, type III DES (HBAs are quaternary ammonium salts, and HBDs cover polyols, carboxylic acids, etc.) are mainly selected as the continuous phase in eutectogels, whose strong hydrogen bond network endows the eutectogels with excellent electrical conductivity, wide temperature range stability and interface adhesion. On the other hand, the sensitivity of the eutectogel-based sensor can be enhanced through the design and construction of a microstructure. Moreover, self-repair and improved mechanical properties of the eutectogels can be achieved by their dynamic networks and multi-network composite collaboration.

Three main mechanisms, i.e., resistance, capacitance, and triboelectricity, for efficient conversion of external stimuli into electrical signals were summarized. The resistance-based sensing directly responds to strain and temperature through changes in the ion migration pathway, and is widely applicable. The capacitance-based sensing uses the eutectogel as the dielectric layer and regulates the capacitance signal through structural deformation, which is suitable for high-precision pressure detection. The triboelectric sensing relies on contact electrification and electrostatic induction coupling to achieve self-powered sensing, breaking the dependence of traditional sensors on external power sources. These characteristics enable the eutectogels to demonstrate significant advantages in various scenarios, such as flexible strain/motion monitoring, microscopic deformation recognition (facial expressions, vocal cord vibration), EMG/ECG signal acquisition, high-precision temperature sensing, and extreme environment detection, making it a key material for promoting the development of flexible sensing technology.

Although eutectogels have effectively expanded the application scenarios of gel-based sensors, there are still many issues that need to be overcome.

Firstly, there is the inherent contradiction between mechanical properties and electrical conductivity and stability. The balance of the three also poses a challenge to the long-term service and durability of sensor devices. The conductive property of the eutectogel depends on the intrinsic electrical conductivity provided by DES. When the mechanical strength is enhanced, the ion migration channels of the eutectogel are blocked, resulting in a lower electrical conductivity. Meanwhile, although some DES containing metal ions provide a large amount of ions, their high viscosity often hinders the ion transmission and the improvement of sensing performance. Therefore, when aiming to achieve high mechanical properties and stability in the eutectogel, while also ensuring excellent sensing performance, new materials and new methods still need to be researched and developed to overcome these challenges.

In the future, by precisely designing dynamic and ionic polymer frameworks to construct dual-network, semi-interpenetrating networks and mixed cross-linked networks, coordinated energy dissipation, functionalization and polymerization can be achieved. The DES was functionalized to inhibit phase separation and volatilization, and nano fillers were incorporated into the eutectogel to improve the mechanical strength and to build a continuous conductive path. This approach can enhance the interfacial compatibility between the gel and DES at the molecular level. Finally, uniform ion transport and stable network were achieved by optimizing the microstructure and polymerization process.

Secondly, the large-scale production of eutectogels is challenging. The synthesis method for eutectogels is complex and requires high precision equipment, meaning that it is difficult to achieve industrial-scale quality control. Moreover, some synthesis methods, such as the solvent substitution method, require a large amount of DES and also involve heating and stirring, which do not meet the requirements of green chemistry and significantly increase the production cost. The uniformity of eutectogels prepared by photopolymerization is difficult to guarantee. The process of forming is difficult, making it challenging to fabricate large-area, ultra-thin, and uniform sensor components.

In the future, by developing industrial-grade low-cost raw materials, constructing continuous photopolymerization molding processes, introducing full-process online quality monitoring systems, and formulating unified preparation and testing standards, the bottlenecks can be overcome from four aspects: raw material cost reduction, process continuity, processing low-temperature compatibility, and quality control standardization. Finally, industrial production of eutectogel sensors can be achieved.

Thirdly, there is no quantitative model between DES, gel structure design and sensing performance. The main approach remains trial-and-error synthesis. At the same time, the calculation methods for parameters such as sensitivity GF, linearity, and hysteresis, which can characterize the performance of the sensor, are all in a state of chaos, and comparisons cannot be made between different papers. This significantly restricts the improvement of the functions of eutectogel sensors and their standardized applications.

In this regard, we established unified testing standards and characterization norms, clearly defining the definitions and calculation methods of core parameters, and standardizing the testing procedures for performance. At the same time, an open and shared performance database and experimental paradigm platform were established to ensure that the data are comparable, repeatable and verifiable, promoting the research on eutectic gel sensing to move from qualitative performance demonstrations to a quantitative scientific system, and providing theoretical and standard support for the practical application and industrialization of the devices.

With the continuous innovation of core technologies and the deepening of interdisciplinary integration, eutectogels will accelerate the process of transforming from laboratory-scale materials to industrial-level technologies. Systematically addressing practical application problems, such as cost control and long-term service stability, will promote their application in scenarios such as precision medicine, smart wearables, and industrial internet of things (IoT). This progress is poised to provide core support for the development of high-performance, multi-functional, and intelligent flexible electronics.

## Figures and Tables

**Table 1:** The selection of DES and key parameter characteristics.

	DES	Eutectogel Composition	Characteristic	Performance Parameter	Advantages	Disadvantages	Applications	References
Selection of DES in eutectogels	Type I ZnCl_2_ ChCl (1:2)	Lignin acrylamide (AM)	Conductivity	1.012 S/m	1. High conductivity 2. Good frost resistance 3. Antibacterial 4. Strong adhesion	1. High viscosity 2. Limited sources	Wound dressings	[27]
			Environmental stability	−90–60 °C				
			Adhesion	Hydrogen bonding Metal coordination Ion–dipole/dipole–dipole interaction				
	Type I ZnCl_2_ ChCl Glycerol (12:7:2)	Hydroxyethyl acrylamide (HEAA)	Conductivity	0.01016 S/m	1. Good frost resistance 2. Antibacterial 3. Strong adhesion	1. High viscosity 2. Limited sources	Wound dressings	[28]
			Environmental stability	-				
			Adhesion	Hydrogen bonding Metal coordination				
	Type II AlCl_3_·6H_2_O AA ChCl (0:2:1, 1:50:25)	Acrylic acid (AA) Cellulose	Conductivity	0.12 S/m	1. High conductivity 2. Good frost resistance 3. Strong adhesion	1. High viscosity 2. Limited sources 3. Unstable	Flexible sensing	[19]
			Environmental stability	−25–60 °C				
			Adhesion	Hydrogen bond Metal coordination Ion–dipole/dipole–dipole interaction				
	Type III ChCl Ethylene glycol(EG) (1:2)	AA	Interfacial impedance	92.2 kΩ at 10 Hz	1. Good interface stability 2. Good frost resistance 3. High transparency	Weak conductivity	Bioelectrode	[29]
			Environmental stability	−80–25 °C				
			Adhesion	Hydrogen bonds Cation–π Microscale interlocking				
	Type III ChCl Glycerol (1:2)	PVA Gelatin	Conductivity	0.67 S/m	1. Good frost resistance 2. High transparency	Long response time	Flexible sensing	[30]
			Environmental stability	−60–25 °C				
			Adhesion	-				
	Type III ChCl Glycerol (1:2)	Sodium alginate(SA)	Conductivity	0.16 mS/m	Excellent environmental stability	1. Weak conductivity 2. Poor adhesion	Flexible sensing	[31]
			Environmental stability	No significant change at 25 °C for 7 days				
			Adhesion	-				
	Type III ChCl AA (1:2)	AM SA	Conductivity	The addition of DES improves the charge transfer ability	1. Anti swelling 2. Antibacterial	1. Weak conductivity 2. Poor adhesion	1. Flexible sensing 2. Temperature alarm	[32]
			Environmental stability	Small change of swelling volume in water				
			Adhesion	-				
	Type III ChCl Urea (1:2)	PVA Gelatin	Conductivity	0.12 S/m	1. Excellent environmental stability 2. Fast response time	Poor adhesion	Flexible sensing	[33]
			Environmental stability	−20–100 °C No significant change at 25 °C for 30 days				
			Adhesion	-				
	Type III Betaine EG (1:3)	MXene HEAA Gelatin	Conductivity	0.56 mS/m	1. Excellent environmental stability 2. Temperature response 3. Strong adhesion	Weak conductivity	1. Flexible sensing 2. Temperature response	[34]
			Environmental stability	−40–40 °C No significant change at 25 °C for 30 days				
			Adhesion	Hydrogen bond Metal coordination				
	Type IV CaCl_2_ Glycerol	Gelatin AM	Conductivity	0.12 S/m	1. Excellent environmental stability 2. Excellent mechanical strength	1. Weak conductivity 2. Poor compatibility with polymer 3. High viscosity	Flexible sensing	[35]
			Environmental stability	−20–60 °C				
			Adhesion	Ion–dipole interactions Dipole–dipole interactions Van der Waals interactions Electrostatic interactions				
	Type IV ZnCl_2_ EG	AA 2-Hydroxyethyl acrylate (HEA) ChCl	Conductivity	4.8 mS/m	1. Antibacterial 2. Good frost resistance	1. Long response time 2. High viscosity	1. Flexible sensing 2. Temperature response	[36]
			Environmental stability	−20 °C				
			Adhesion	Hydrogen bonds Cation–π Dipole–dipole interactions				
	Type IV ZnCl_2_ EG (1:2, 1:3, 1:4, 1:5, 0:1)	PVA AA PDA	Conductivity	1.6 S/m	1. Temperature response 2. Excellent environmental stability 3. high sensitivity	Poor compatibility with polymer	1. Flexible sensing 2. Photothermal therapy	[37]
			Environmental stability	No significant change at 25 °C for 30 days				
			Adhesion	Electrostatic Hydrogen bonding, π–π Cation–π interactions				
	Type V Menthol Thymol	Poly(benzyl acrylate) (PBnA)	Type V non-ionic eutectic solvent has inherent defects such as poor compatibility with hydrogel, strong plasticization effect and strong hydrophobicity. At present, it can only be used as an auxiliary plasticizer or hydrophobic modifier, and its application in the sensing field is still greatly limited.				Dissolved polymer	[38]

**Table 2:** The design of eutectogel networks and their key parameter characteristics.

Element	Method	DES	Eutectogel Composition	Feature	Performance Parameter	Advantages	Disadvantages	Applications		References
Microstructure design	Mold transfer method	ChCl Glycerol (1:2)	Gelatin	Hierarchical spinous microstructures	Sensitivity(S): 6.16 kPa^−1^ (0–22 kPa), 1.70 kPa^−1^ (22–99 kPa), 0.34 kPa^−1^ (99–173 kPa)	Ultra-high sensitivity	1. Poor mechanical properties 2. Complex preparation process	Tactile sensing	[39]	
	3D printing	ChCl EG (1:2)	N-hydroxymethyl acrylamide (NAM) Hydroxyethyl methacrylate (HEMA)	Pyramid microstructure	Sensitivity(S): 0.615 kPa^−1^ (0–0.7 kPa)	Ultra-high sensitivity	Complex preparation process	1. Breath detection 2. Vocal cord vibration sensing	[40]	
Dynamic network design	Introduction of dynamic crosslinking group	Citric acid Diallyldimethylammonium chloride (1:1, 1:2, 2:1, 1:3, 3:1)	Glycidyl methacrylate-modified gelatin	Hydrogen bond Electrostatic interaction	Self-healing efficiency of 72.83% after 48 h	Improving the service life of eutectogel	Performance cannot be fully recovered	Flexible sensing	[41,42]	
		ChCl EG (1:2)	PVA Gelatin Carboxymethyl cellulose sodium	Hydrogen bond Schiff base	Self-healing efficiency of 80% after 1 h	1. High self-healing efficiency	Performance cannot be fully recovered	Tactile sensing		
Multi network and composite design	Introduction of conductive material	CaCl_2_ ChCl Glycerol	PVA SA PEDOT:PSS	PEDOT:PSS	Gauge Factor = 1.41	Excellent electrical conductivity	Poor compatibility between conductive material and matrix	1. Flexible sensing 2. Temperature response	[43]	
	Introduction of photothermal responsive materials	ChCl AA (1:2)	PVA SA MXene	MXene	Different conductivity at different temperatures	1. Temperature response 2. Antibacterial	Mxene is prone to oxidative deterioration	Flexible sensing	[44]	
		Lithium bis(trifluoromethanesulfonyl)imide (LiTFSI) ZnCl_2_	N-isopropylacrylamide(NIPAM) vinyl acetate (VA)	NIPAM	TCR of −77.18% °C^−1^ (R^2^ = 0.996) from −10 to 6 °C, −25.50% °C^−1^ (R^2^ = 0.970) from 6 to 15 °C, and −10.96% °C^−1^ (R^2^ = 0.984) from 15 to 25 °C	Thermal safety management	Limited temperature detection range	1. Flexible sensing 2. Thermal safety management	[45]	
		ChCl Glycerol (1:2)	Poly(vinyl pyrrolidone) (PVP) Tannic acid(TA) Acrylamide (AM)	Fe_3_O_4_	1. Low detection limit, 0.5% 2. Rapid response time (0.83 s) 3. Frost resistance (−40 °C)	Color-changing effect	1. Vision electric signal dual-mode complementary sensing 2. Controllable performance and excellent linearity	Insufficient performance during long-term service	[46]	

**Table 3:** The composition, sensing mechanism, performance parameters and applications of eutectogels.

Components of DES	Molar Ratio	Eutectogel Composition	Sensing Mechanism	Core Properties	Core Parameter	Applications	Ref.
AA ChCl AlCl_3_·6H_2_O	1:2:0.0005 1:2:0.0009 1:2:0.002	AA	Resistive sensing	Tensile property	3200 ± 200%	Bioelectrode	[19]
				Self-adhesive	52.1 kPa to glass		
ChCl AlCl_3_·6H_2_O Glycerol	50:1:100	PAA PVA Lignin	Resistive sensing	Conductivity	10 mS/cm	1. Flexible strain sensing 2. Low temperature sensing	[14]
				Frost resistance	−20 °C		
ChCl ZnCl_2_ Glycerol	2:0.2:1	N-hydroxyethylacrylamide (HEAA)	Resistive sensing	Compressive strength	19.280 MPa	1. Flexible strain sensing 2. Wound dressing	[28]
				Antibacterial	-		
ChCl EG	1:2	Zitterionic monomer 3-dimethyl(methacryloyloxyethyl)ammonium propanesulfonate (DMAPS) AA	Resistive sensing	Low Young’s modulus	2.6–17.3 kPa	1. Flexible strain sensing 2. Bioelectrode	[29]
				Conductivity	0.1 S/m		
				Adhesion	155 kPa to porcine skin		
ChCl EG	1:2	PVA Borax AgNWs	Resistive sensing	Conductivity	3.937 S/m	1. Flexible strain 2. Microscopic deformation sensing	[118]
				Self-healing performance	Recover to 75% of the original strength within 10 min.		
ChCl LiCl EG	1:1:3	Crboxymethylcellulose (CMC) Dodecylbenzene sulfonic acid (DBSA) PEDOT	Resistive sensing	Conductivity	0.87 mS/cm	1. Flexible strain 2. Microscopic deformation sensing	[53]
				Self-adhesion	11 kPa		
				Tensile properties	610%, 81 kPa		
ChCl AA Urea	1:2:2	PVA	Resistive sensing	Gauge factor	1.95 for the strain range of 0–30%	Microscopic deformation sensing	[119]
Sorbitol Citric acid	2:3	Gelatin	Resistive sensing	Conductivity change	950%	1. Microscopic deformation sensing 2. Breath detection	[120]
				Detection range	22–98% RH		
ChCl EG Urea	-	AA Polyvinylpyrrolidone (PVP) modified by Pyramid microstructure	Resistive sensing	Minimum detection limit	1 Pa	Microscopic deformation sensing	[121]
ChCl Glycerol	1:2	AA Konjac glucomannan	Resistive sensing	Adhesion	84.5 kPa to skin	EMG and ECG signal sensing	[122]
				Low impedance	235.8 kΩ, 1 Hz		
				Frost resistance	−40 °C		
ChCl Glycerol	1:2	Waterborne polyurethane (WPU) Tannic acid (TA)	Resistive sensing Piezoresistive	Adhesion	12.5 N/m to skin	EMG and ECG signal sensing	[1]
				Conductivity	0.22 mS/cm		
				Sensitivity	284.4 kPa^−1^		
Vinylphosphonic acid ZnCl_2_	4:1	Lithium bis(trifluoromethanesulfonyl)imide (LiTFSI) N-isopropylacrylamide (NIPAM)	Resistive sensing	Ultra-high temperature coefficient of resistance (TCR)	−77.18% °C^−1^	Temperature sensing	[45]
				Wide operating temperature range	−10–120 °C		
AA Glycerol	-	AA Polyaniline nanofibers (PANI NFs)	Resistive sensing	GF	18.28	Temperature sensing	[123]
				Thermosensation	−0.016 °C^−1^		
				Temperature resolution	2.7 °C		
Phytic acid(PA) ChCl	20:1 10:1 5:1 3:1	PVA PA	Resistive sensing	Frost resistance	−40 °C	Extreme environment sensing	[62]
Lactic acid(LA) ChCl	9:1	Glycidyl methacrylate (GMA)	Triboelectric sensing	Wide operating temperature range	−40–100 °C	Extreme environment sensing	[117]
ChCl EG H_2_O	1:3:3	AA	Resistive sensing	Frost resistance	Low resistance (131.4 Ω) at −20 °C	Extreme environment sensing	[124]

## Data Availability

No new data were created or analyzed in this study. Data sharing is not applicable to this article.
